# From Anticipatory Fear to Professional Appreciation: A Cross-Cultural Observation of Emotional Experiences in Medical Students During Initial Donor Dissection

**DOI:** 10.1007/s40670-026-02936-9

**Published:** 2026-10-26

**Authors:** Jickssa M. Gemechu, Cameron J. Davidson, Abay M. Zenebe, Abdo R. Jurjus, Eiman A. Meguid, Inaya Hajj-Hussein

**Affiliations:** 1 Oakland University William Beaumont School of Medicine, 586 Pioneer Dr, Rochester, MI 48309 USA; 2 Addis Ababa University, Addis Ababa, Ethiopia; 3 American University of Beirut, Beirut, Lebanon; 4 Queen’s University Belfast, Northern Ireland, UK

**Keywords:** Donor dissection, Anatomy education, Cultural attachment, Coping strategies, Emotional experience, Medical students, Cross-cultural

## Abstract

Human donor dissection is a formative psychological experience in medical education that elicits diverse emotional, cognitive, and behavioral responses. The influence of cultural context on students’ emotional experiences, anxiety, and coping strategies remains insufficiently understood across geographically and culturally diverse settings. This study examined and compared these psychological responses among first-year medical students before and after their initial dissection experience. First-year medical students from four institutions: Oakland University William Beaumont School of Medicine (USA), the American University of Beirut (Lebanon), Queen’s University Belfast (Northern Ireland), and Addis Ababa University (Ethiopia) completed surveys one week before (T1) and one week after (T2) their first dissection. Data were analyzed using one-way analysis of variance with Bonferroni correction. Emotional experiences differed significantly among institutions at T1 but converged at T2, suggesting that the dissection experience itself brought students from different cultural backgrounds to a similar emotional level. Cultural attachment remained stable across time points. Anxiety decreased at T2 across all institutions. Positive coping showed no significant differences at T1 but differed significantly at T2, while negative coping differed significantly at both time points. These findings indicate that donor dissection produces largely adaptive psychological responses across culturally diverse medical student populations, reinforcing its value as a pivotal shared experience in professional identity formation. The stability of cultural attachment suggests that it shapes how students interpret and emotionally process dissection rather than being altered by it. These results support the development of anatomy curricula that are evidence-based and responsive to the cultural background of their students.

## Introduction

Anatomy is one of the oldest and most fundamental disciplines in medical science, with human donor^1^ dissection serving as a cornerstone of anatomical education since the 12th century [1, 2]. Despite the growing integration of advanced imaging technologies, digital simulations, and virtual dissection tools, donor dissection continues to play a vital role in many medical curricula worldwide due to its unmatched value in cultivating tactile, spatial, and clinical understanding of the human body [3–7]. Through direct interaction with a human donor, students develop a nuanced appreciation of anatomical variation, structural relationships, and clinical relevance, foundational competencies for future clinical practice. This direct encounter with the human body extends well beyond the acquisition of anatomical knowledge alone, engaging students in an experience that is simultaneously emotionally, morally, and professionally transformative.

First-year medical students’ initial encounter with a human donor represents a formative psychological event that can elicit a wide spectrum of emotional responses. These include shock, anxiety, discomfort, sadness, and distress, intensified by the unfamiliar environment, the smell of preservatives, and the visceral reality of working with a human body for the first time [8–10]. Alongside these negative emotional reactions, many students simultaneously report positive responses such as curiosity, gratitude, and a sense of professional privilege, reflecting the dual nature of this experience as both emotionally challenging and professionally formative [11, 12]. The intensity and quality of these emotional responses are not uniform across medical students; they are shaped by individual psychological traits, prior exposure to donors, institutional support structures, and the broader sociocultural context in which learning takes place [13–15]. Among these responses, anxiety is particularly well documented from a psychological and cognitive arousal triggered by the perceived threat or novelty of the dissection environment [16, 17]. It is most pronounced prior to first exposure, during a period of anticipatory appraisal, and tends to attenuate following actual contact with the donor, as the stressor becomes more familiar and manageable [18–20]. Physical manifestations of anxiety, including nausea, dizziness, and palpitations, have also been reported, though these too tend to diminish as students adapt over time [21, 22]. Understanding the antecedents and trajectory of anxiety is therefore essential for designing pre-dissection preparation that effectively reduces unnecessary distress while preserving the pedagogical value of the experience.

Cultural attachment, defined as the psychological connection individuals maintain with their cultural values, spiritual beliefs, and social norms, in this case regarding the human body, death, and post-mortem practices, adds a further layer of complexity to the dissection experience [23]. Drawing on cultural psychology frameworks [24–26], students from collectivist cultural settings may process the encounter through communal, relational, or religious frameworks, while those from more individualist contexts may rely on personal coping repertoires and individual appraisal. In cultures where the deceased body is revered, dissection may be experienced as spiritually unsettling or morally transgressive, whereas in others it may be broadly accepted yet still emotionally taxing due to conflicting familial or religious expectations [4, 27–30]. Importantly, cultural attachment may function not merely as a contextual backdrop but as a stable psychological moderator that influences how students appraise and respond to dissection, shaping both the quality of initial emotional reactions and the coping trajectories that follow.

Coping strategies represent the behavioral and cognitive mechanisms through which students regulate the emotional demands of dissection. Drawing on Gross’s [31] process model of emotional regulation, students may employ antecedent-focused strategies such as cognitive reframing, preparation, and distraction prior to dissection, or response-focused strategies such as seeking peer support, engaging in reflective practice, or participation in institutional rituals following the experience. Common approaches reported in the literature include emotional detachment, compartmentalization, and viewing the body as a learning tool, as well as more prosocial strategies such as group discussion and donor memorial ceremonies [32–39]. The effectiveness and accessibility of these strategies vary considerably across students, and culturally sensitive interventions such as integrating narrative reflection and emphasizing donor gratitude have been shown to meaningfully reduce distress and support professional growth [14, 32, 40–42]. Whether students adopt predominantly positive or negative coping strategies has meaningful implications for their long-term professional well-being and adjustment to clinical training.

This multicenter study examines the interplay of emotional experience, anxiety, cultural attachment, and coping strategies among first-year medical students at four institutions representing distinct geographic and cultural contexts: Oakland University William Beaumont School of Medicine (OUWB, United States of America), the American University of Beirut (AUB, Lebanon), Queen’s University Belfast (QUEENS, Northern Ireland), and Addis Ababa University (AAU, Ethiopia). By assessing students at two time points surrounding their initial donor dissection experience, one week before and one week after, the study measures changes in psychological and emotional responses across this formative event. Findings are discussed in light of established stress-appraisal, emotional regulation, and cultural psychology frameworks, with the aim of advancing a more nuanced understanding of how medical students navigate the psychological demands of donor dissection across culturally distinct educational settings.

## Materials and Methods

Participants in this study were first-year medical students taking an Anatomy course at four institutions: OUWB, AUB, QUEENS, and AAU. A total of 525 students were included in the study. Participants from the first three institutions completed the survey online, whereas those from the fourth institution (AAU) completed it in person, which contributed to the larger number of participants.

### Design and Procedure

This study employed a longitudinal, pre-post survey design to explore medical students’ perceptions, emotional experiences, cultural attachments, and coping strategies related to donor dissection. Data were collected using a survey administered at two time points: Time 1 (T1), a week before initial exposure to donor dissection experience, and Time 2 (T2), one week after the first dissection experience. This design allowed for evaluation of within-subject and between-group changes across the three domains of interest.

### Survey Instrument

The survey instrument was adapted from a previously validated study conducted at OUWB School of Medicine [43] and structured into four sections: (A) demographic information, including age, gender, religion, ethnicity, and prior dissection experience; (B) the dissection experience using a modified *Appraisal of Life Events (*ALE) Scale; (C) self-reported stress and anxiety Indicators; and (D) coping strategies assessed using the *Brief COPE* inventory. The instrument was reviewed for content validity by subject matter experts in anatomy education and cross-cultural psychology. Ethical approval was obtained from the institutional review board at each participating institution, and informed consent was secured from all participants prior to data collection. Participant recruitment announcements were sent through email (OUWB, AUB, QUEENS) or announced in class (AAU).

### Measures

#### Emotional Experience and Anxiety (ALE Scale)

Emotional experience was operationalized using the Appraisal of Life Events (ALE) Scale (Ferguson et al., 1999), a validated instrument that assesses subjective appraisal of stressful events across three dimensions: threat, challenge, and loss. The scale comprises 14 emotion-based items rated on a Likert scale, spanning both negative responses (e.g., scary, nauseating, anxiety-provoking) and positive responses (e.g., stimulating, enjoyable, interesting). Anxiety-related items within the scale specifically capture physiological and cognitive arousal responses, including nausea, sleep disturbance, and general distress. Students completed the ALE Scale at both T1 and T2 to capture changes in emotional appraisal across the dissection experience. Prior work supports the reliability and construct validity of this measure in medical students exposed to donor dissection [9, 44].

#### Cultural Attachment

Cultural attachment was measured using a 4-item scale assessing students’ psychological connection to their cultural values and beliefs, specifically in relation to their views on the human body, death, and post-mortem practices [23]. Items evaluated cultural identity, the personal salience of cultural beliefs, and whether those beliefs influenced the student’s experience in the dissection room. Responses were aggregated into a composite score and subsequently dichotomized into high versus low cultural attachment based on the 50th percentile. Internal consistency, as measured by Cronbach’s alpha, ranged from borderline to acceptable between the time points (T1: α = 0.61; T2: α = 0.71).

#### Coping Strategies

Coping was assessed using the Brief COPE Scale [45], a 28-item instrument comprising 14 subscales that measure distinct behavioral and cognitive coping strategies: active coping, planning, positive reframing, acceptance, self-distraction, religious coping, behavioral disengagement, self-blame, seeking instrumental support, seeking emotional support, venting, substance use, humor, and denial. Each item is rated on a 4-point scale from 1 (minimal use) to 4 (frequent use). For the present analyses, subscales were grouped into positive coping strategies (e.g., active coping, positive reframing, acceptance) and negative coping strategies (e.g., denial, behavioral disengagement, self-blame). The Brief COPE has been extensively validated in healthcare and academic settings [46].

### Statistical Analysis

Survey data were exported from Qualtrics and analyzed using IBM SPSS Statistics (Version 28.0.0). Response rates differed significantly between the two time points, so they were subjected to individual one-way analysis of variance (ANOVAs) and follow-up post-hoc analyses for each variable to maximize sample size for each school at each time point. Bonferroni adjustments were applied to control for Type I error associated with multiple comparisons. Grand means (collapsed across schools) for T1 and T2 were analyzed using Welch-corrected independent t-tests to assess the overall effect for time. A significance level of *p* < 0.05 was established a priori for all statistical tests. Graphs were created with GraphPad Prism (Version 10.6.1).

## Results

This study evaluated and compared students’ self-reported emotional experiences, the influence of cultural attachment on these emotions, and the coping strategies they adopted across four institutions with differing curricula at two time points surrounding the donor dissection experience.

### Demographic Profile

A total of 525 first-year medical students from four institutions participated in the study: from OUWB (*n* = 56), AUB (*n* = 81), Queens (*n* = 78), and AAU (*n* = 310). Although gender distribution varied by institution, overall representation of male and female students was relatively reflective of the slightly larger number of female students. Age distributions differed: most students at AAU and Queens were under 20 years of age, while the majority of students at AUB and OUWB were older, reflecting differences in admission routes and educational systems. Across all four institutions, the participants reported a religious affiliation, ranging from 79% at OUWB to 97% at AAU. Prior exposure to donor dissection was uncommon overall, with less than 5% having experience and all instances came from the AAU site (Table 1).

**Table 1:** Demographic distribution of respondents across four institutions (OUWB most common age (MCA) = 23; AUB MCA = 21; Queens MCA < 20; AAU MCA < 20) by gender, age, religious affiliation, and prior dissection experience. The percentage for each variable reflects the portion of total sample with missing data represented

Characteristics	Category	OUWB (*n* = 56)	AUB (*n* = 81)	Queens (*n* = 78)	AAU (*n* = 310)
Gender	Male	18 (32%)	37 (46%)	19 (24%)	154 (50%)
	Female	34 (63%)	42 (52%)	58 (74%)	155 (50%)
Age distribution	≤ 20	0 (0%)	23 (28%)	68 (87%)	298 (96%)
	> 20	56 (100%)	58 (72%)	10 (13%)	11 (4%)
Identifying with any religion	Yes	44 (79%)	68 (84%)	62 (80%)	302 (97%)
	No	12 (21%)	11 (14%)	16 (21%)	7 (2%)
Previous experience with a donor dissection	Yes	0 (0%)	0 (0%)	0 (0%)	16 (5%)
	No	56 (100%)	81 (100%)	77 (99%)	291 (94%)

The most commonage (MCA) for each institution is as follows: OUWB = 23; AUB = 21; Queens = 20; AAU = <20. This is organized by gender, age, religious affiliation,and prior dissection experience. The percentage for each variable reflects the proportion of the total sample with missing data represented

The demographic distribution by ethnic background and religious affiliations varied considerably across the four institutions, reflecting the cultural diversity of the study population. Participants from AAU students identified as Amhara (54%), Oromo (17%), or Tigre (4%), whereas AUB students were predominantly Arab (98%). Queens students reported a more diverse range of backgrounds, including Irish (45%), British (32%), South Asian (9%), and Middle Eastern (7%). The OUWB cohort primarily consisted of White Americans (52%), Asian Americans (21%), and Middle Eastern students (11%). Regarding religious affiliations, Christian affiliations formed the majority at AAU (84%), Queens (68%), AUB (51%), and OUWB (59%), while Muslim students represented a substantial proportion (31%) at AUB (Table 2).

**Table 2:** Ethnic background and religious affiliations of respondents across four institutions

Institution	Predominant ethnic background	Other reported groups	Predominant religious affiliation	Other reported groups
OUWB (USA)	White Americans (52%)	Asian American, Middle Eastern, African American, Hispanic/Latino, Others	Christian (59%)	Muslim, Jewish, Hindu, Others
Queens (Northern Ireland)	Irish (45%)	British, South Asians, Middle Eastern, Others	Christian (68%)	Muslim, Buddhist, Hindu, Other
AUB (Lebanon)	Arab (98%)	Armenian, others	Christian (51%) Muslim (31%)	Others
AAU (Ethiopia)	Amhara (54%)	Oromo, Tigre, others	Christian (84%)	Muslim, Others

The findings of this study revealed significant differences among students from different institutions in their self-reported emotional experiences, cultural attachment, anxiety-provoking experiences, and positive and negative coping strategies at two time points.

### Emotional Experiences

**Fig. 1:**
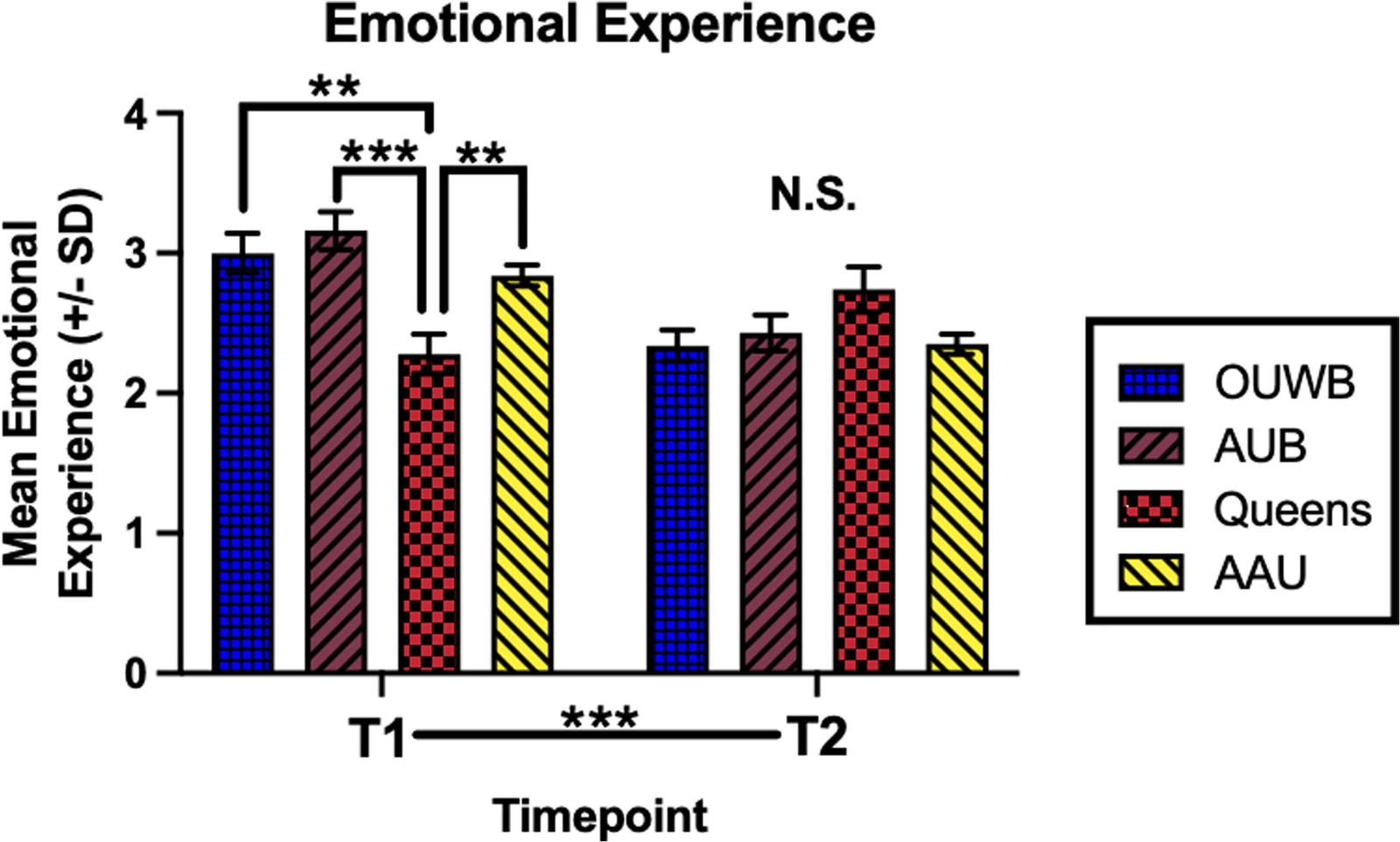
Emotional experience responses from four medical schools (OUWB, AUB, Queens, and AAU). The responses revealed significantly lower scores for Queens compared to the other three medical schools at T1 only. OUWB, AUB, and AAU were not significantly different from one another at T1 and there were no significant differences between schools at T2. Analysis of mean differences between T1 and T2 across the combined sample showed a significant reduction in mean emotional experience (*p* < 0.001). Note; Significance stars on the figure represent significance levels of *p* < 0.05 (“*”); *p* < 0.01 (“**”); *p* < 0.001 (“***”)

Emotional experience response manifested significant differences among the four student groups at T1, with students at QUEENS reporting the lowest level (x̅ =2.28), followed by AAU students (x̅ =2.84), and those in OUWB & AUB x̅=3.00 and x̅= 3.16, respectively (F = 7.11; ^*^p<0.001). The four groups did not show any significant differences on this measure at T2 (see Fig. 1). When comparing the overall difference between T1 and T2, there was found to be a significant overall reduction in mean Emotional Experiences t(1013) = 5.369, *p* < 0.001.

### Anxiety-Provoking Experiences

**Fig. 2:**
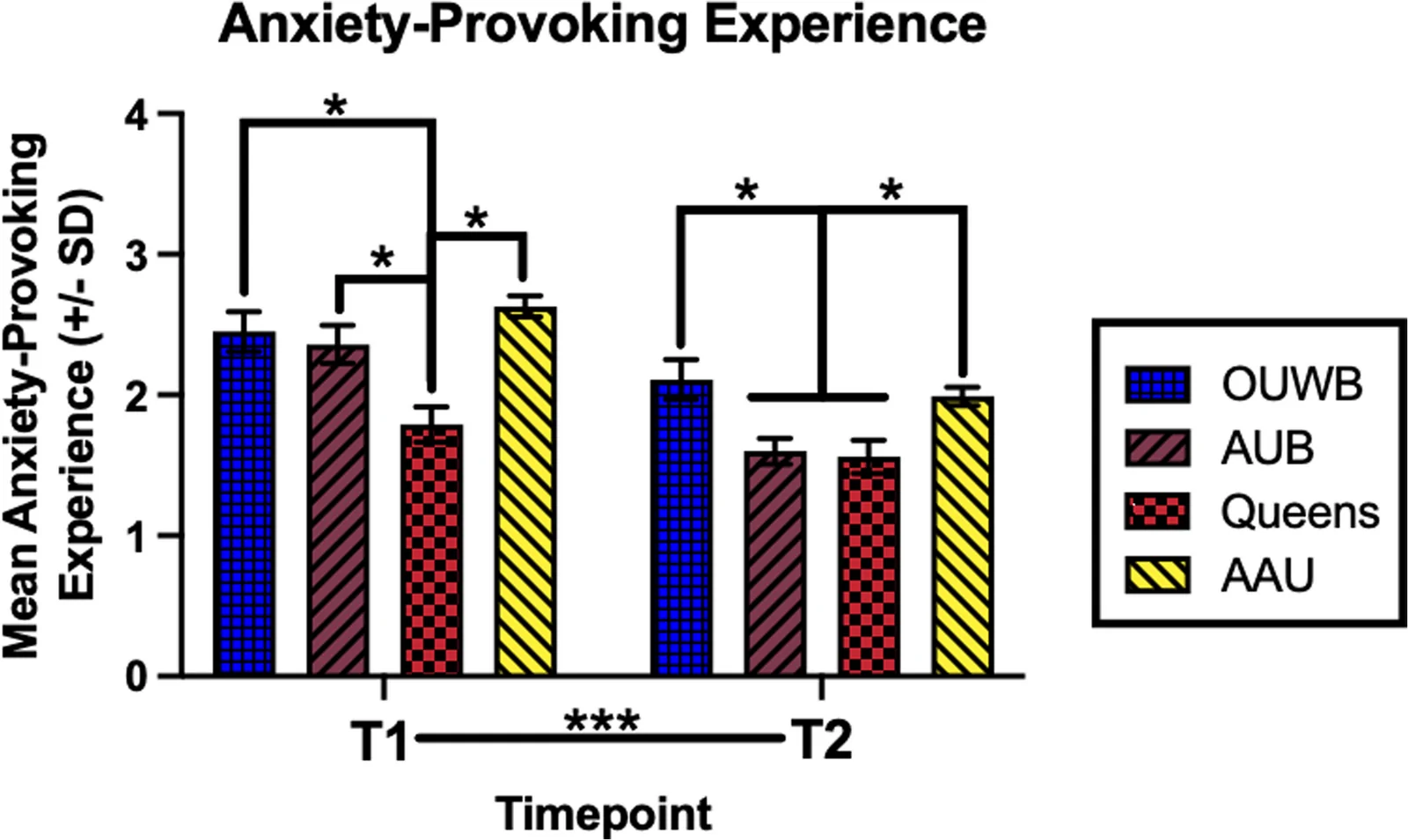
Anxiety-provoking experience response from four medical schools (OUWB, AUB, Queens, and AAU). The responses revealed significantly lower scores for Queens and the other three medical schools at T1, with the other three not showing significant differences from one another. Within T2, OUWB and AAU both had significantly higher Anxiety-provoking experiences than AUB and Queens. Analysis of mean differences between T1 and T2 across the combined sample showed a significant decline in Anxiety-provoking experience, t(1002) = 7.391, *p*<0.001. Note; Significance stars on the figure represent significance levels of *p* < 0.05 (“*”); *p* < 0.01 (“**”); *p* < 0.001 (“***”)

Anxiety-provoking experience response manifested significant differences among the four student groups at both T1 and T2, with students at QUEENS showing the lowest scores at T1 (x̅ =1.79), compared to AUB (x̅ =2.36), OUWB (x̅ =2.45), and AAU (x̅ =2.63); F = 9.52; ^*^p<0.001. At T2, anxiety scores decreased across all four schools with QUEENS (x̅ =1.56), AUB (x̅ =1.60) significantly lower than those of AAU (x̅=1.99), and OUWB (x̅ =2.11). Analysis of mean differences between T1 and T2 across the combined sample showed a significant reduction of Anxiety-Provoking experiences t(1002) = 7.391, *p* < 0.001 (see Fig. 2).

### Cultural Attachments

**Fig. 3:**
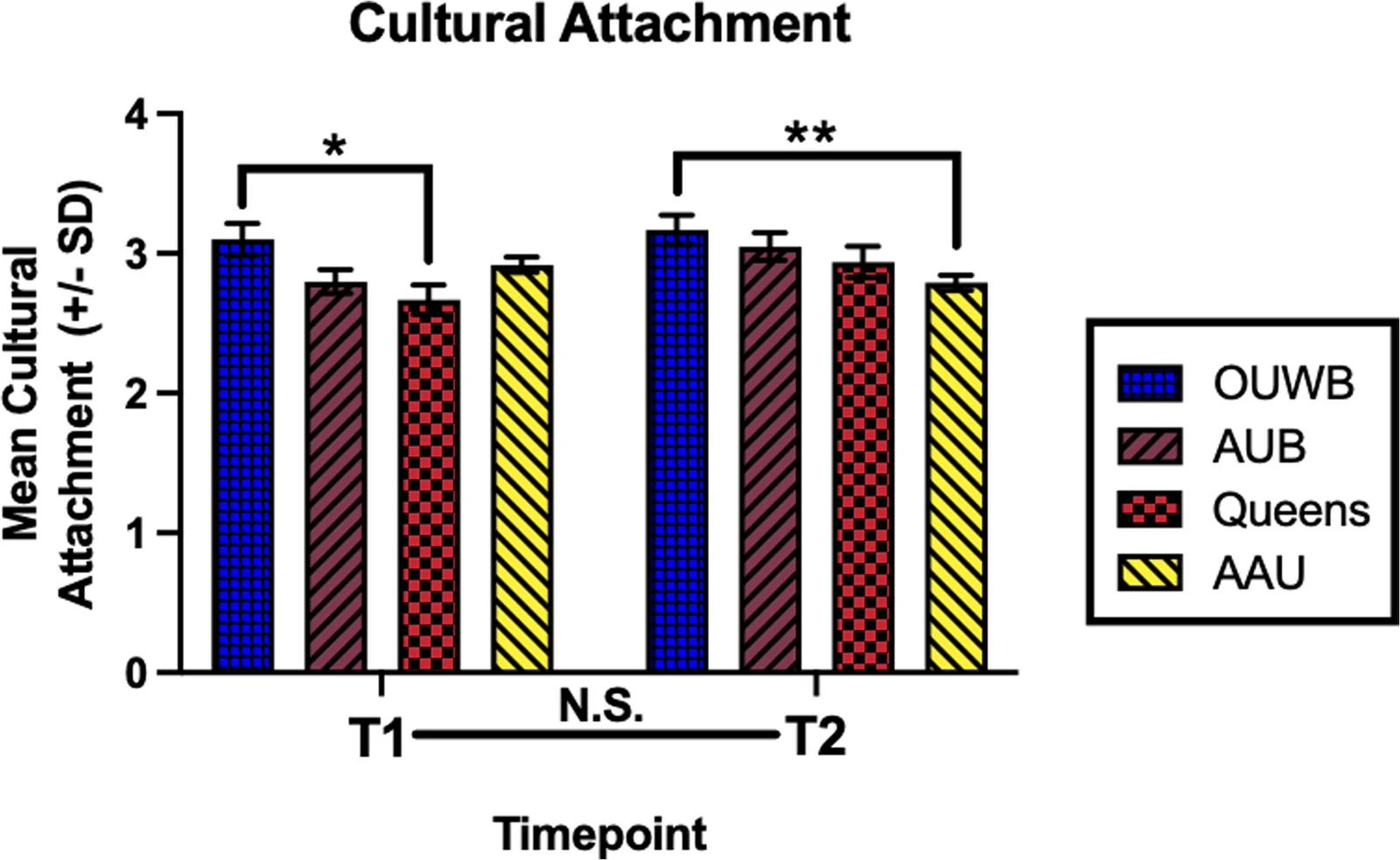
Cultural attachment responses from four medical schools (OUWB, AUB, Queens, and AAU). The responses revealed a significant difference between OUWB and Queens at T1 and OUWB and AAU at T2. Analysis of mean differences between T1 and T2 across the combined sample showed no significant difference, indicating relative stability for this metric. Note; Significance stars on the figure represent significance levels of *p* < 0.05 (“*”); *p* < 0.01 (“**”)

Cultural attachment responses manifested significant differences among the four student groups, with OUWB students reporting the highest levels at T1 (x̅ = 3.10), followed by students in AAU (x̅ = 2.92), students at AUB (x̅ = 2.80), and QUEENS (x̅ =2.67); F = 2.72; ^*^p<0.05; (See Fig. 2). The only significant difference was between OUWB and Queens at T1 (*p* < 0.05). Within T2, OUWB remained the highest but was only significantly different from AAU (*p* < 0.01). Analysis of cultural attachment showed no significant change from T1 to T2 t(1014) = 0.3035, *p* = 0.762, suggesting that it may function as a stable trait at least over the short term. As such, it may serve as a predictor of other emotional responses and coping strategies related to donor dissection, rather than being influenced by them (see Fig. 3).

### Coping Strategies

Positive and negative coping strategies showed distinct patterns across schools and time points. Within positive coping strategies, the groups did not differ at T1, however, OUWB and AAU were significantly higher than AUB and Queens at T2. Analysis of mean differences between T1 and T2 across the combined sample for positive coping demonstrates a significant increase in positive coping at T2 t(798.2) = 2.080, *p* < 0.05.

**Fig. 4:**
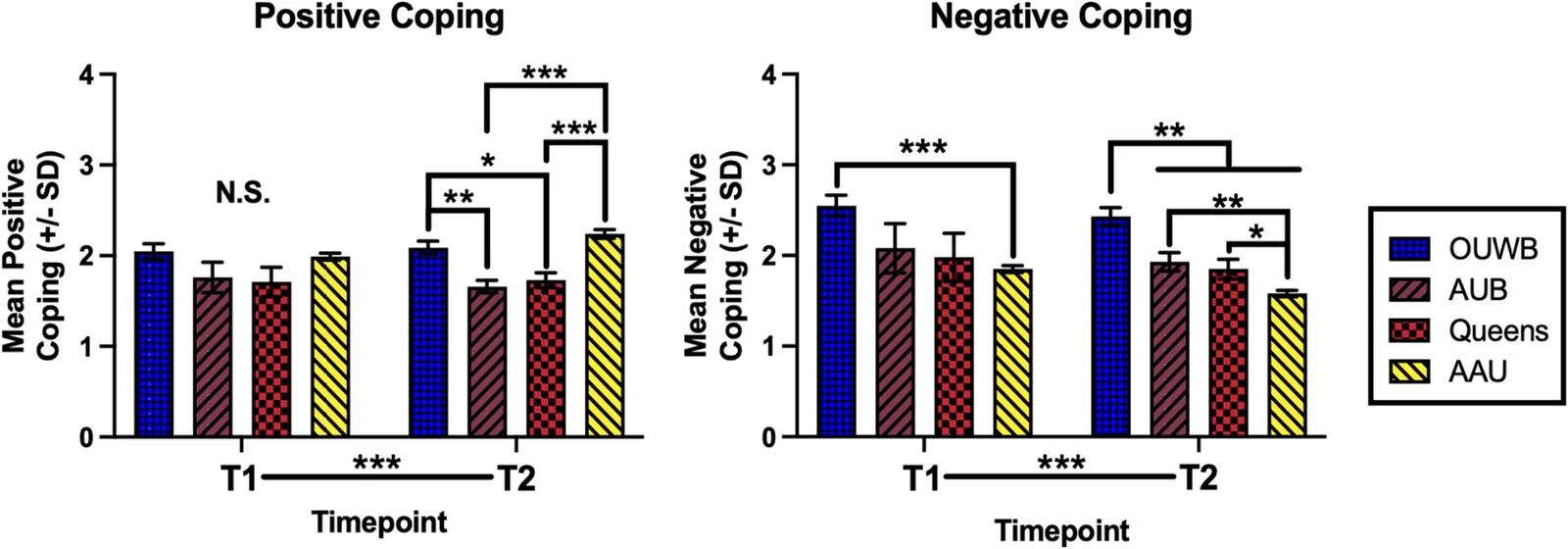
Coping strategy response from four medical schools (OUWB, AUB, Queens, and AAU). The responses revealed significant differences between the four medical schools at T1 and T2 for negative coping but not in T1 for positive coping. Analysis of mean differences between T1 and T2 in the entire sample (all four medical schools combined) showed a significant difference for both positive (increase; *p*<0.05) and negative coping (decrease; *p*<0.01). Note; Significance stars on the figure represent significance levels of *p* < 0.05 (“*”); *p* < 0.01 (“**”); *p* < 0.001 (“***”)

Within negative coping, OUWB demonstrated significantly higher levels of negative coping than AAU at T1. At T2, OUWB demonstrated significantly higher levels of negative coping than the 3 other universities (*p* < 0.01). Additionally, AUB and Queens were significantly higher than AAU (*p* < 0.05) but did not differ from each other (See Fig. 4). Analysis of mean differences between T1 and T2 across the combined sample demonstrated significantly less negative coping following donor experience t(767) = 2.374, *p*<0.01.

### Summary of Time Point Comparisons

Analysis of mean differences between T1 and T2 in the entire sample (all four schools combined) showed no significant change in cultural attachment; however, a significant reduction in emotional experience, anxiety-provoking and negative coping, but an increase in positive coping. These findings demonstrate a positive emotional progression following a single donor dissection experience without impact on cultural attachment.

## Discussion

The majority of studies examining medical students’ emotional and psychological responses to donor dissection have been conducted within a single country or institution, limiting the generalizability of their findings to specific cultural and educational contexts. Studies originating from the United States reflect a predominantly individualist cultural framework, while those conducted in the Middle East, Africa, or the United Kingdom operate within distinct religious, communal, and professional socialization environments that fundamentally shape how students appraise stress, regulate emotion, and develop coping strategies in response to the dissection experience. To our knowledge, no prior study has systematically examined these psychological responses including emotional experience, anxiety, cultural attachment, and coping strategies across institutions selected for their geographic and cultural heterogeneity. This multicultural, multicentered design is the defining strength of the present study. This design allows for a direct comparison of how cultural context moderates students’ psychological trajectory through this formative event, rather than treating culture as a background variable.

Our findings demonstrate a range of emotional responses to this formative experience, with the most pronounced differences between institutions observed at T1. Importantly, an overall convergent trend emerged following the dissection, suggesting that the experience itself may operate as a homogenizing factor across cultural contexts. Our work builds on and extends from earlier research examining cultural attachment in this setting [47, 48].

Emotional experience differed significantly between institutions at T1, which seems to be partly attributed to cultural affiliation. Queens students reported the lowest emotional experience prior to donor dissection. Following the dissection, an overall downward shift from T1 to T2 was seen across all institutions, with between-institution differences largely attenuating. This convergence suggests that the dissection experience itself may exert a homogenizing effect on emotional responding and supports the interpretation that anticipatory emotional experience is substantially shaped by cultural and cognitive factors. These include uncertainty and culturally mediated beliefs about death and the human body that diminish once students encounter the structured reality of the dissection environment. This is consistent with prior work demonstrating that actual emotional distress during dissection is typically lower than students anticipated, regardless of cultural background [9, 19, 49].

Anxiety showed a similar but not identical pattern. Queens students again reported significantly lower anxiety than the other three institutions at T1, and this difference was maintained at T2 rather than converging as it did for emotional experience. A general decline in anxiety from T1 to T2 was observed across institutions; however, OUWB and AAU remained significantly higher than AUB and Queens at T2, indicating that anxiety reduction was not uniform across cultural contexts. These differential trajectories highlight the need for further investigation into the institutional, cultural, and pedagogical factors that moderate anxiety adaptation following first donor dissection [19, 20].

Cultural attachment was robust to the first dissection experience, showing no significant change from T1 to T2 across any institution. This stability suggests that cultural attachment may function as a relatively constant trait through which other emotional and coping responses are filtered, rather than being itself modified by the dissection encounter. Important to note is that OUWB reported the highest level of cultural attachment overall, significantly different from Queens at T1 and AAU at T2. This may reflect broader patterns of cultural identification among American medical students relative to other institutions. These findings warrant further investigation into the stability of cultural affiliation across the full arc of medical training and across a wider range of cultural contexts [13, 36, 50].

An instructive pattern emerged when examining coping strategies. For positive coping, no between-institution differences were present at T1, which is conceptually coherent given that students had not yet had the experience to cope with. Following dissection, a significant increase in positive coping was observed at OUWB and AAU, while AUB and Queens showed comparatively little change, resulting in significant between-group differences at T2. The negative coping data added further nuance: OUWB reported significantly higher negative coping than AAU even at T1, and remained significantly elevated relative to all three other institutions at T2. The overall reduction in variance from T1 to T2 across institutions is consistent with the interpretation that T1 responses partly reflected speculative or anticipatory appraisals, while T2 responses reflected more grounded post-experience assessments. Taken together, OUWB students demonstrated the highest relative use of both positive and negative coping strategies throughout, which may reflect genuine cultural differences in emotional expressivity, greater social acceptability of acknowledging and articulating emotional responses, or both [9, 19, 20, 49].

The broader emotional landscape of the dissection experience is also shaped by the institutional and national policy contexts within which it occurs. In traditional Western anatomical sciences education, policies of donor anonymity were designed to facilitate clinical detachment. A professional emotional distance intended to enable students to engage objectively with the donor; however, this approach was associated with elevated initial anxiety and, when unsupported, risk of cynicism [51]. In response, many Western institutions have moved toward humanizing practices including donor life stories and memorial services, which appear to channel anticipatory anxiety into professional empathy and motivation [52]. In Middle Eastern contexts, cultural and religious reverence for the deceased informs students’ emotional framing of the experience; Lebanese students, for example, have been found to experience significant emotional impact while drawing on distinct social support mechanisms compared to their American counterparts [43]. In African institutions, the historical reliance on unclaimed bodies introduces a distinct form of moral distress that is increasingly addressed through structured faculty guidance and ethical workshops designed to cultivate gratitude and professional responsibility [53]. Across all these contexts, the transition from anticipatory fear to professional appreciation is substantially dependent on whether institutional policy positions the donor as a humanistic figure whose contribution is honored, thereby transforming potential distress into a foundation for professional identity development.

The overall improvements observed from T1 to T2 can be explained by an experiential learning framework. Within Kolb’s model, the dissection experience operates as a salient “concrete experience” that initiates a cycle of reflection, cognitive restructuring, and behavioral adaptation [54]. Emotionally charged experiences are recognized as potent drivers of learning precisely because they disrupt prior expectations and necessitate the integration of new cognitive and affective information. In the context of anatomy education, the anticipatory anxiety and uncertainty observed at T1 is well documented and reflects not only culturally mediated beliefs about death, and the human body, but also the compounding anxieties of a new academic environment, unfamiliar curricula, and the broader transition of entering medical school. The post-dissection shifts towards less anxiety and improvements in adaptive coping likely reflect the transformation of this uncertainty into structured understanding, facilitated by reflective processing, faculty normalization, and social support within the learning community. The collective nature of the dissection experience may additionally foster intra-cohort bonding through shared engagement with a challenging event, potentially producing a form of mild post-traumatic growth as students accomplish the task systematically within a supportive peer and faculty environment. Empirical work in medical education consistently demonstrates students report initial reactions of shock, fear, and anxiety prior to the first dissection experience, followed by relatively rapid emotional adaptation [10, 32]. The initial survey of experiences before the dissection occurred likely reflects the uncertainty of the situation, perceived threat, and culturally mediated beliefs about death and the human body. This transition suggests that the dissection experience provides an opportunity for students to reconcile their preconceived fears with the structured and purposeful nature of the learning environment. Such changes are consistent with the literature drawing from anxiety and phobia therapy that encourages persons to directly engage with previously avoided stimuli. These parallel experiences seem to promote cognitive reappraisal and cognitive adaptation, particularly when this occurs in a controlled and supportive context like the anatomy dissection laboratory in a medical school curriculum.

Examining this further, the findings are consistent with exposure-based models of anxiety reduction. Exposure-based therapy, which is typically seen as the first-line intervention for phobias and anxiety-related disorders, operates through repeated, safe confrontation with the offending stimuli inducing cognitive, psychosocial, and interoceptive acclimation to this over time [55]. Exposure paradigms suggest that repeated meaningful contact with this stimulus can reduce avoidance and increase psychological flexibility, thus allowing individuals to engage more openly with this as a previously distressing concept [56]. In the current context, donor dissection may function analogously to a single-session exposure in which students confront multi-sensory stimuli associated with death, bodily integrity, and cultural taboos (although cultural attachment in our study was unimpacted by this experience). Particularly of note, is that our results demonstrate a very mild change in response to this single session which is in line with evidence that emphasizes that anxiety reduction is not solely attributable to within-session habituation. Rather, the process of inhibitory learning, adaptation, and fear extinction, whereby new, non-threatening associations are formed and compete with the prior fear-based expectations [57] takes place over time and with multiple sessions. Qualitative studies support this interpretation, documenting a characteristic shift from distress towards more complex emotional responses, including curiosity, professional responsibility, and emerging professional identity [32]. Importantly, exposure effects are typically strengthened when the actual experience violates prior expectations, as appears to be the case here, given that anticipated distress at T1 consistently exceeded reported distress at T2. This can facilitate deeper learning and longer retention of new associations. This indicates that the findings of the current study would likely be amplified with repeated exposures across the medical education curriculum. These could represent a mild or transient experience that would be reversed; however, the co-occurrence of shifts in coping and reductions in anxiety fit well within the framework as individuals experience the normalized environment and learn that the socio-emotional response is tolerable and does not necessitate protection in the form of anxiety or avoidance. This reflects the idea that this is a foundational formative experience that contributes to identity formation and the internalization of attitudes towards death, empathy, and respect for the human body [21]. Taken together, the improvements observed one week post-dissection likely reflect a broader cognitive-affective reorganization, in which students integrate the emotional, cultural and professional significance of the dissection experience into more adaptive coping frameworks.

## Conclusion

This multicultural, multicentered study provides evidence that the first donor dissection experience produces consistent and largely adaptive psychological changes across geographically and culturally diverse medical student populations. While between-institutions differences in emotional experience, anxiety, and coping were most pronounced before the dissection, these differences attenuated substantially following the experience, underscoring the capacity of structured anatomical sciences education to transcend cultural boundaries as a shared professional milestone.

Cultural attachment, by contrast, remained stable throughout, suggesting it functions less as an outcome of the dissection experience and more as a stable personal characteristic. These findings support the integration of experiential learning principles and exposure-based pedagogical strategies into anatomy curricula via donor dissection across diverse institutional contexts. This also reinforces the view that the first dissection is not merely a technical milestone but a foundational event in the formation of professional identity, empathy, and attitudes toward death and the human body. Understanding how cultural context shapes, but does not determine, this trajectory is essential for designing anatomy education curricula that are both scientifically grounded and culturally responsive.

These conclusions should be considered in light of several methodological limitations, including the reliance on self-reported measures, the assessment of responses to a single dissection session within a one-week period, and variation in dissection content across institutions. Future studies incorporating objective measures, longer follow-up periods, and more standardized exposure protocols would help validate and strengthen these findings (see limitations and future directions section below).

## Limitations and Future Directions

The study has some limitations to consider. Sample sizes were unequal across institutions, with AAU contributing over half of the total sample. This may impact the balance and thus the interpretation of between-group comparisons. The two time point data collection window captures only immediate pre- and post-dissection responses and does not allow for assessment of longer-term emotional adaptation across the curriculum. All outcome measures rely on self-report, introducing potential social desirability and recall bias, which may be particularly pronounced in cultural contexts where public acknowledgement of distress carries social stigma. Prior dissection experience varied across institutions, ranging from approximately 5% at AAU to 0% at all other institutions. This was not statistically controlled in the primary analyses, which may confound between-group baseline comparisons. Finally, the absence of qualitative data limits the depth of interpretation, with further rationale and/or context not able to be added from the student’s own perspective and findings may be difficult to generalize beyond the four participating institutions.

Future longitudinal designs tracking students across multiple dissection encounters and into clinical training are needed to determine whether the adaptive changes observed at T2 are sustained or amplified with repeated exposure. Incorporating qualitative and mixed-methods components would capture how students make sense of the dissection experience and emotional adaptation that follows. Expanding the study to include institutions from a wider range of geographic and cultural contexts beyond those represented here would substantially strengthen the cross-cultural generalizability of these findings.

## Data Availability

The datasets generated and analyzed during the current study are available upon request.

## Footnotes

1 Throughout this article, the term ‘human donor dissection’ is used instead of ‘cadaver dissection’ to show appropriate deference and emphasize the altruism and humanity of individuals who made the choice to donate their body for education and research.
